# The Symptoms and Treatment of Inflammation of the Ciliary Body (Acute Cyclitis.)

**Published:** 1876-01

**Authors:** 


### Translations.

### THE SYMPTOMS AND TREATMENT OF INFLAMMATION OF THE CILIARY BODY (ACUTE CYCLITIS.)

FROM A. MOOREN'S SYMPATHETIC DISTURBANCES OF VISION.

Translated from the German, by F. C. HOTZ, M.D.

It is a remarkable fact that, however easy may be the diagnosis of cyclitis, the profession is as yet but little familiar with an exact knowledge of the nature of this malady.

Besides the photophobia and lachrymation, one of the first signs of incipient cyclitis is a well marked circle of vascular injection around the cornea. This vascular zone surrounding the cornea like a circle composed of radiating lines, was well known to the ancients under the name of the arthritic vascular ring, and is now known to be common in all forms of acute keratitis and iritis as well as in the first stage of granular conjunctivitis. This symptom alone, therefore, would not be sufficient to establish the existence of cyclitis ; it does not acquire any diagnostic significance until it is associated with a great tenderness to pressure of the ciliary region. Pressure with the tip of the finger upon the closed eyelids will never fail to produce a pain which all patients agree in localizing in the upper and inner portion of the globe ; but the severity of this pain differs according to the extent of the inflammation and the individual degree of sensibility. It is not unusual to see a robust workman who, under other circumstances, would bravely endure the pain of any external lesion, groan and faint from that caused by a gentle pressure upon the eyelids. Sometimes the sensitiveness is so acute that patients can not tolerate the lightest bandage over their eyes. And wherever we suspect an inflammation of the ciliary ring, we can discover the starting point and seat of the inflammation—latent though it be—with an absolute certainty, by passing a probe over the ciliary region, as I

have frequently seen it done by Von Graefe. There is no symptom more certain and undeceptive for the surgeon as well as for the patient.

The symptoms just enumerated are soon followed by an increase in the depth of the anterior chamber. This phenomenon is less due to an increased secretion of aqueous humor than to a retraction of the iris caused by inflammatory adhesions of its periphery to the ciliary processes. This morbid process is manifested by a projection of the ligamentum pectinatum* which encases the periphery of the iris as the frame does the crystal of a watch.

In very grave cases I have seen this projection developed in from 20 to 24 hours after a contusion of the ciliary region. Even when the retraction of the iris extends over its whole circumference, the pupil sometimes shows a faint mobility ; and is always with comparative facility dilated by atropia unless the iris is implicated in the inflammation. The complete retraction of the iris is an extremely dangerous obstacle to the circulation ; for the arteries of the iris, supplied by the two long posterior ciliary and branches of the anterior ciliary arteries, pass over into veins at the pupillary margin and can no longer empty their blood into the venous ring of the ciliary processes and the vasa vorticosa. In consequence of this impeded circulation, the veins of the iris appear engorged and tortuous, the epithelial layer is veiled by a fine mist, the aqueous humor becomes turbid ; and with these symptoms we generally observe a slight hypopyon that comes and goes. If the cyclitis does not yield to the treatment, or the retraction of the iris has existed for a longer time, slight effusions of blood into the anterior chamber are not of rare occurrence, and the ocular conjunctiva, previously somewhat puffy, soon exhibits the character of a chemosis.

* Ligamentum pectinatum is the name given to the fibrous attachment of the anterior surface of the iris with Descemet's membrane; its origin is a little behind the margin of the transparent cornea.

The impairment of the sight keeps pace with the aggravation of the malady. The ophthalmoscopic examination, however, proves that the disturbance of vision is not due merely to the turbidity of the aqueous humor, but should be, to a great extent, attributed to the rapid formation of opacities filling the anterior portion of the vitreous humor, now in the form of a dense mist and again in the shape of numerous floating flakes.

These local symptoms are always attended by signs of constitutional disturbances, usually those of gastric disorder with more or less febrile excitement.

Such are the essential symptoms of cyclitis, but one or another may predominate at times ; sometimes the vascular injection, and the symptoms of engorgement that result therefrom, develop with unusual severity ; sometimes the neuralgic pain prevails most persistently, resisting the largest doses of quinine, or, by extension, it invades the whole tri-facial nerve, leaving a sensation of numbness over the corresponding half of the face and head, during the period of remission.

In other cases again, the inflammatory and neuralgic symptoms are comparatively slight ; the increased secretion constitutes the prominent symptom ; and in consequence of the unusual depth of the anterior chamber, the greater width of the pupil and the fine deposits upon the posterior surface of the cornea, the disease greatly resembles serous iritis. We could not affirm that this or that series of symptoms resulted from specific influences ; it seems, however, to be a matter of fact, that cyclitis supervening upon operations — that for cataract, for example—sometimes even iridectomy, has a great tendency to assume the character of a purulent inflammation. This tendency to suppuration is still more pronounced in cases of violent contusions of the ciliary region. Under these circumstances a purulent infiltration of the cornea nearly always coexists with or is dependent upon the cyclitis ; and terminates in complete necrosis of that membrane.

Whatever form the cyclitis may exhibit, the sensitiveness of the ciliary ring to the touch and the presence of opacities in the vitreous humor, are constant symptoms. I have observed but two cases in which the character of the malady was less distinctly defined, and in which even a transient cloudiness of the vitreous humor did not take place, although there was the greatest tenderness on pressure, a most violent neuralgia which followed each attempt at accommodation, and a most vivid pericorneal redness.

Cyclitis, even when spontaneous, is undoubtedly one of the most dangerous inflammations that can befall the eye; for, besides the importance of the ciliary body itself, the malady, often insignificant and scarcely perceptible in the beginning, may become the source of the most formidable danger.

My observation of the fact, that every attempt of the sound eye at accommodation increases the pain in the eye affected by cyclitis, has induced me to begin the treatment by covering the sound eye with a bandage. After instilling a solution of atropia (Atrop. sulph. gr. ij; Aq. dest. ℥j), I ordered the inflamed eye to be covered by warm poultices, to which hyoscyamus or any other narcotic drug, as herba conii or capita papaveris, may be added, in order to increase—as many physicians suppose—the sedative properties of the poultices. I can not say that I have found any difference in the effect of simple poultices and those containing narcotic medicaments. But especial attention was given to having the poultices of a uniform and moderately warm temperature, and to their being renewed at once as soon as the patient complained of chilliness.

In extremely painful cases, the poultices were continuously applied during 4, 6, 10 and even 24 hours, and reapplied after the patient had rested for awhile. This treatment, so simple and yet so beneficial, has never been varied; only where we have had to deal with much chemosis, the use of the poultices was preceded by the application of leeches to the inner canthus. The leeching had to be repeated very seldom, and was done in those cases only where the robust constitution of the patient did not contra-indicate its employment.

Besides the use of atropia, which acted as a sedative on the secretory nerves of the eye, morphine was administered at night and the hypodermic injections employed as often as the intensity of the symptoms urgently required their use. Hence it has happened that the hypodermic injections had to be repeated twice or three times during a day, although morphine was given regularly every evening. As soon as the neuralgia assumed an intermittent type, quinine was given in large doses. If it did not show any intermittent character, we preferred to prescribe magnesiæ sulph. with sodæ vitr., unless the gastric disorders indicated the use of sodæ bicarb. or the acids. Whenever the cyclitis was accompanied by increased secretion of aqueous humor, the derivatives were discontinued and diuretic remedies substituted ; among which the extr. colocynth. combines the derivative and diuretic action most happily. In general, we did not rely upon one remedy exclusively ; scilla maritim. was used, as well as digitalis, or coccionella. Of late years, we have ceased to employ sudorific remedies, not only because with most patients they increase congestion of the head and thereby aggravate the malady indirectly, but also because the constant perspiration renders the patient too liable to catch cold. As a rule, we abstain in cyclitis from any operative interference wherever it can possibly be avoided ; we perform paracentesis of the cornea only in rare cases and never do iridectomy, no matter how turbid the aqueous humor nor how copious the hypopyon. For, under these conditions, the operation is uncertain in its results, and may be followed by very disastrous consequences.

Such are the simple rules which guide me in the treatment of cyclitis. In cases only of wounds of the sclerotic, or of coexisting irido-choroiditis, the use of atropia is strictly prohibited, because experience has taught me that the use of atropia under such conditions invariably aggravates the malady. Nor is the atropia used in cases distinguished by excessive vascular injection with chemosis of the conjunctiva, until a decided decrease of the severe injection is observed.

I have no hesitation in saying, that this mode of treatment has proved to be as efficacious as it was simple; for the subsidence of the pain usually was the first sign of its favorable influence; subsequently, a decrease of the vascular injection occurred, while the aqueous humor became clear, and the hypopyon disappeared. I have seen recent cases of frightful cyclitis, in which the entire periphery of the iris was retracted, recover completely within fourteen to eighteen days by the continued use of poultices and a few instillations of atropia.

However favorable a course the disease may take if submitted to rational treatment from the beginning, we must avoid becoming too hopeful. I have seen the neuralgia persist for weeks in spite of the use of the most powerful narcotics, in spite of the most careful attendance, and in spite of all imaginable precautions observed by the patient. At this moment I have under my care a patient suffering from acute granular ophthalmia complicated by cyclitis; during five entire months he had been tortured by the most violent pain day and night. The largest dose of morphine taken internally had no effect worth mentioning; the hypodermic injections only brought relief for a few hours; but complete intermission did not occur till within the last weeks. In this case a most salutary influence on the cyclitis may be attributed to the occurrence of a favorable change in the weather; for at that time the vitreous humor began to clear up, and a few posterior synechiæ were the sole remaining signs of a coexisting iritis.

